# Acute Labyrinthitis Revealing COVID-19

**DOI:** 10.3390/diagnostics11030482

**Published:** 2021-03-09

**Authors:** Marie Perret, Angélique Bernard, Alan Rahmani, Patrick Manckoundia, Alain Putot

**Affiliations:** 1 Geriatric Internal Medicine Department, Centre Hospitalier Universitaire Dijon Bourgogne, 21000 Dijon, France; marie.perret@chu-dijon.fr (M.P.); patrick.manckoundia@chu-dijon.fr (P.M.); 2 Radiology Department, Centre Hospitalier Universitaire Dijon Bourgogne, 21000 Dijon, France; angelique.bernard2@chu-dijon.fr (A.B.); alan.rahmani@chu-dijon.fr (A.R.)

**Keywords:** labyrinthitis, inner otitis, hearing loss, vertigo, COVID-19, SARS CoV2, MRI

## Abstract

An 84-year-old man presented to the emergency department for acute vomiting associated with rotational vertigo and a sudden right sensorineural hearing loss. A left peripheral vestibular nystagmus was highlighted. The patient was afebrile, without respiratory signs or symptoms. Blood sampling at admission showed lymphopenia, thrombopenia and neutrophil polynucleosis, without elevation of C reactive protein. Cerebral magnetic resonance imaging eliminated a neurovascular origin. Vestibule, right semicircular canals and cochlear FLAIR hypersignals were highlighted, leading to the diagnosis of right labyrinthitis. A nasopharyngeal swab sampled at admission returned positive for SARS CoV2 by polymerase chain reaction. The etiologic investigation, including syphilitic and viral research, was otherwise negative. An oral corticotherapy (prednisone 70 mg daily) was introduced, followed by a progressive clinical recovery. Although acute otitis media have already been highlighted as an unusual presentation of COVID-19, radiology-proven labyrinthitis had to our knowledge, never been described to date.

In mid-November 2020, at the epidemic peak of the first COVID-19 wave in France, an 84-year-old man presented to the emergency department for acute vomiting associated with rotational vertigo in the standing position. He also reported a sudden right sensorineural hearing loss. The patient did not complain of any abdominal pain or pulmonary symptoms.

His medical history included arterial hypertension treated by LOSARTAN 50 mg per day and an aneurysm of the abdominal aorta and of iliac arteries, treated by endovascular stent and for which a long-term anticoagulant (WARFARINE 2 mg/day) treatment was required.

On arrival, the patient was afebrile, with a blood pressure of 110/78 mm Hg, and a pulse of 84 beats/minute. The room air oxygen saturation was at 99% and a respiratory rate of 20/min without respiratory signs or symptoms. Lung auscultation was normal.

Laboratory tests at admission showed a white blood cell count at 2.8 × 10^3^/mm^3^ with lymphopenia at 0.4 × 10^3^/mm^3^/mm^3^ normal neutrophil polynucleosis count at 2.57 × 10^3^/mm^3^/mm^3^, and platelet count at 118 × 10^3^/mm^3^. C reactive protein was measured at 20 mg/L. Liver and kidney functions were both within the normal ranges.

We performed a complete initial physical examination, looking for specific symptoms that would point us towards one of the possible causes of vertigo [1]. We highlighted spontaneous, bilateral, horizontal twitching of the eyes with rapid movements to the left, indicating a left peripheral vestibular nystagmus. No deficits or other signs of neurological focus were found. The continuous character of the vertigo did not evoke a Meniere’s disease.

Brain magnetic resonance imaging excluded neurovascular neoplastic disease. However, vestibule, semicircular canals and cochlear on the right appeared hyperintense on FLAIR images (Figure 1) and on diffusion-weighted images, leading to the diagnosis of right labyrinthitis.

Viral research including HSV, VZV, HIV, CMV, hepatitis B, hepatitis C, as well as syphilitic serology, were negative. Clinical examination excluded otitis media or meningitis as a cause of secondary suppurative bacterial labyrinthitis. Anti-nuclear antibodies, anticytoplasmic neutrophil antibodies, anti-MPO, anti-PR3, anti-MBG antibodies were also negative.

Finally, a nasopharyngeal swab sampled at admission returned positive for SARS CoV2 by reverse transcription polymerase chain reaction (Aptima^®^ SARS-CoV-2 Assay, Hologic, Marlborough, MA, USA).

High signal intensity on FLAIR magnetic resonance imaging with normal T1 sequences, as presented in this case report, is a radiological pattern specific to an acute inflammatory process (i.e., acute labyrinthitis) [2,3]. Labyrinthitis is an inflammation of the membranous labyrinth. It can be caused by viruses, bacteria, or systemic disease [4]. Even though we cannot exclude a coincidental finding, in the absence of other etiology, we concluded that this acute labyrinthitis was very likely due to COVID 19, even in the absence of classical COVID-19 symptoms. Indeed, viruses responsible for upper respiratory tract infection are the most common cause of labyrinthitis [4]. Proposed mechanisms include direct viral invasion, reactivation of a latent virus within the spiral ganglion, and an immune-mediated mechanism in a systemic viral infection [5]. Currently, the damaging impact of COVID-19 virus on the hearing organs in the inner ear is a new finding yet to be explored [6,7,8]. Otitis has already been highlighted as an unusual presentation of COVID-19, in conjunction with usual viral symptoms or as an isolated disorder [8,9,10,11,12].

The patient was admitted at the epidemic peak of COVID-19, presented a brutal asthenia one week before and regressive lymphopenia and thrombopenia at admission, suggesting that acute labyrinthitis occurred at the acute phase of COVID-19 [13]. A comparative study of the amplitude of transient evoked otoacoustic emissions and thresholds of pure-tone audiometry between asymptomatic COVID-19 PCR-positive cases and normal non-infected subjects suggests that cochlea involvement in COVID-19 could be frequent, even without major symptoms [14]. However, radiology-proven labyrinthitis had to our knowledge, never been described to date.

In the absence of respiratory symptoms, no specific COVID-19 treatment was recommended. Because of the labyrinthitis, a one-week oral corticosteroid therapy (prednisone 70 mg daily) was started, associated with physiotherapy rehabilitation, followed by a progressive clinical recovery. Corticosteroid has proven efficiency in acute vestibular vertigo [15]. However, there is to date very limited data concerning corticotherapy usefulness in COVID-19 related otitis. Treatment with oral corticotherapy with or without intratympanic dexamethasone injections has been proposed [10,12] with progressive clinical improvement in most of cases.

## Figures and Tables

**Figure 1:**
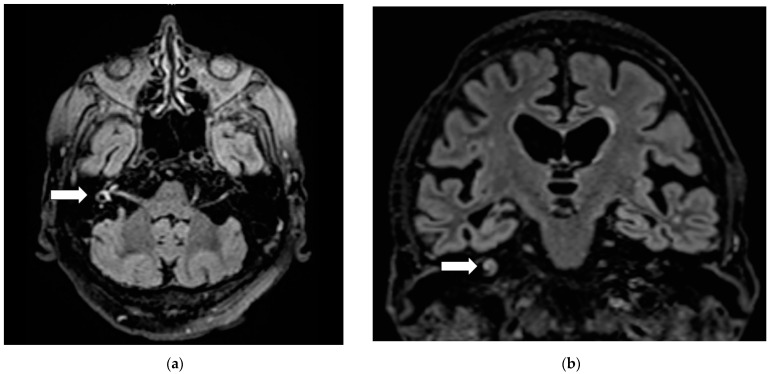
Right labyrinthitis: Hyperintensity (arrows) on axial (**a**) and frontal (**b**) FLAIR sections in right vestibule, semicircular canals and cochlear.

## Data Availability

Data supporting reported results are available from the corresponding author on reasonable request.
